# Evidence Synthesis Gone Awry: The Perils of Aggregating Ineffective or Unsafe Doses in Alopecia Areata Reviews

**DOI:** 10.7759/cureus.102230

**Published:** 2026-01-24

**Authors:** Arya Babul, Devina Mehta, Yssra Soliman, Momina Hussain, Najib Babul

**Affiliations:** 1 Biomedical Sciences, Society for Awareness of Neglected Diseases, Las Vegas, USA; 2 Biomedical Sciences, West Career and Technical Academy, Las Vegas, USA; 3 General, Surgical, and Cosmetic Dermatology, Thomas Dermatology, Las Vegas, USA; 4 Genomics, Chinese Academy of Tropical Agricultural Sciences, Sanya, CHN; 5 Drug Development, Quadra Therapeutics, Las Vegas, USA

**Keywords:** alopecia areata, baricitinib, deuruxolitinib, dose selection, evidence synthesis, jak inhibitors, network meta-analysis, ritlecitinib, safety signals, unapproved doses

## Abstract

Network and conventional meta-analyses can increase precision for clinical decision-making but risk producing misleading hierarchies when they pool ineffective, unsafe, or unapproved dosing regimens alongside licensed therapies. Using recent evidence syntheses in alopecia areata, we show how inclusion of small, underpowered dose strata and regimens that never advanced to pivotal trials or were not pursued for regulatory approval (for example, deuruxolitinib 4 mg twice‑daily {BID}, deuruxolitinib 12 mg BID, and ritlecitinib 200 mg loading doses) can distort pooled efficacy and safety estimates, elevate unapproved regimens in rankings, and invite inappropriate causal inferences. We outline key methodological safeguards and offer concrete recommendations as follows: prespecify exclusion or planned sensitivity analyses for unapproved doses, transparently report approval status and relevant regulatory actions (for example, clinical holds) alongside pooled safety estimates, avoid causal attributions from small strata and present uncertainty appropriately, and report sensitivity analyses that omit unapproved doses. Implementing these practices preserves the statistical advantages of meta-analysis while protecting clinicians, guideline panels, and payors from misleading inferences drawn from small or unrepresentative dose groups.

## Editorial

*The combination of studies does not remedy the defects of the individual studies.* - Sir Austin Bradford Hill, *Principles of Medical Statistics* (1937) *[1]*

Introduction

Pivotal registration trials are typically randomized, placebo‑controlled (RCTs) and often lack active comparators. When multiple promising agents within the same pharmacologic class are available, the absence of head‑to‑head trials generates clinical uncertainty regarding their relative effectiveness. When a new drug enters a familiar pharmacologic class, it often evokes both interest and caution. For some clinicians, it signals a promising innovation and invites early adoption. For others, unfamiliarity with dosing, adverse effects, or long‑term safety prompts understandable reticence. These divergent responses reflect broader patterns in the adoption of new treatments, with early adopters emphasizing potential advantages, while late adopters often await accumulated clinical experience, post‑marketing surveillance, or guideline endorsement before incorporating new agents into practice.

In this context, tools for quantitative evidence synthesis, including meta‑analysis and network meta‑analysis, can support clinician decision‑making, guideline development, formulary and reimbursement decisions, and future research prioritization. However, reliable recommendations require rigorous assessment of homogeneity, independence, transitivity, and consistency.

Some meta-analyses sidestep more foundational concerns as follows: incorporating investigational or approved drugs at doses known to be ineffective or unsafe, equating agents approved for unrelated indications with those rigorously vetted by major regulatory bodies for the target condition, and comparing monotherapy with combination regimens (e.g., background topical therapy) without transparent disclosure or appropriate statistical adjustment. We briefly examine three recent dermatology evidence syntheses that include the Janus kinase inhibitor (JAKI) deuruxolitinib, currently approved in the United States for severe alopecia areata (AA) [2].

AA is a common, organ‑specific autoimmune disorder characterized by acute or chronic nonscarring hair loss in well‑defined areas of the scalp, face, and body, including the eyebrows, eyelashes, beard, pubic, and axillary regions, with a reported global prevalence ranging from approximately 0.1% to 2% [3,4]. Severe scalp involvement, defined as ≥50% hair loss based on the Severity of Alopecia Tool (SALT) score, is frequently associated with substantial psychological distress, social isolation, and economic burden, affecting not only individuals but also their families and cohabitants [5-8].

Oral JAKIs have markedly advanced the therapeutic landscape for AA. Before their emergence, treatment relied largely on off-label pharmacologic agents, used as monotherapy or in combination despite a paucity of high-quality evidence [9-15]. Such treatments frequently provide only partial remission, and relapses remain common [16,17]. In contrast, oral JAKIs have been evaluated in rigorously designed clinical trials, signaling a shift toward evidence-based therapeutic development in dermatology [2,18-24].

Baricitinib, a JAK1/JAK2 inhibitor, and ritlecitinib, a JAK3/TEC (tyrosine kinase expressed in hepatocellular carcinoma) inhibitor, received regulatory approval for the treatment of severe AA in major markets, including the United States, United Kingdom, European Union, China, and Japan, beginning with baricitinib’s landmark U.S. approval in mid‑2022, followed by ritlecitinib in mid‑2023. Together, these agents have transformed AA management by offering targeted systemic therapies that modulate the underlying autoimmune pathology [18-24]. However, clinically meaningful scalp hair regrowth (defined as SALT ≤20) is achieved by only about one‑third of patients after 24 weeks, with more than half failing to reach this threshold even after 48 or 52 weeks of uninterrupted therapy [2,18-28]. Treatment discontinuation frequently results in loss of benefit, and some responders fail to recapture their prior level of efficacy upon reinitiation [29,30].

Interindividual variability in response, coupled with the chronic nature of AA and the need for safer, long‑term therapies, underscores an unmet need [26,31-33]. Moreover, all approved JAKIs carry FDA black‑box warnings (or their equivalent in other major regulatory markets) for serious infections, malignancy, major adverse cardiovascular events (MACE), and thrombosis [2,21,22].

Given these limitations, dermatologists have welcomed the recent availability of deuruxolitinib as an additional JAKI option for severe AA. Deuruxolitinib, a deuterated JAK1/JAK2‑selective inhibitor, received FDA approval in mid‑2024 but entered the United States market only in mid‑2025 following resolution of an intellectual property dispute [2]. Its approval was supported by the THRIVE‑AA1 and THRIVE‑AA2 phase 3 trials conducted across the United States, Canada, and Europe, which demonstrated robust efficacy relative to placebo [2,23,34]. Deuruxolitinib exhibits greater inhibitory potency against JAK1, JAK2, and TYK2 compared with JAK3, although the clinical relevance of selective inhibition across JAK, TEC, or TYK family enzymes in AA remains uncertain [2,21,22].

Although baricitinib and ritlecitinib have been available for several years to treat severe AA, clinical experience with deuruxolitinib remains limited due to its recent introduction. Not unexpectedly, no head‑to‑head RCTs compare these agents, and uncertainty persists regarding their relative effectiveness and the provisional hierarchical preferences.

Meta‑analysis can increase precision and statistical power by pooling results from independent randomized trials to estimate whether a drug is superior to a placebo and whether different doses yield distinct outcomes. Network meta‑analysis extends this approach by enabling simultaneous estimation of relative effects among multiple interventions using a common comparator, typically a placebo, when direct comparisons are lacking. Importantly, the pivotal registration trials for baricitinib, ritlecitinib, and deuruxolitinib all included placebo arms, facilitating both conventional and network meta‑analytic comparisons.

A meta-analysis assumes that pooled studies address the same clinical question using commensurable effect measures, with small or explainable between-study differences and adequate, unbiased study selection. To confirm robustness, sensitivity analyses are essential. Network meta-analysis adds further requirements, such as transitivity and consistency, a connected network, and clearly defined intervention nodes. Unlike many investigator-sponsored trials, these standards are routinely met in industry-sponsored global registration studies, even when not optimally described in publications. Such studies are typically designed, executed, and audited by large multidisciplinary teams operating under international guidelines for good clinical (GCP), manufacturing (GMP), laboratory (GLP), and statistical practice (GSP), and are subject to strict regulatory compliance audits. These drug development teams are further supported by an array of external subject matter experts and then undergo robust review by highly experienced multidisciplinary teams at regulatory agencies.

However, despite best intentions, our reporting practices for meta-analysis and network meta-analysis often fall short of the rigor expected of quantitative evidence synthesis, particularly when methodological tools are applied without the judgment they require. This editorial does not aim to critique individual publications; rather, we highlight a few issues of particular relevance to clinical pharmacologists, dermatologists, and other stakeholders involved in the therapeutic management of AA. For instance, Kalantan et al. recently published a dose-ranging meta-analysis involving 1,372 randomized patients treated with deuruxolitinib or placebo for AA [35]. Notably, the manuscript was submitted after key data, some of which diverged from the authors’ evidence synthesis and conclusions, had already become publicly available, with critical evidence accessible well before submission.

The 4 mg, 8 mg, and 12 mg twice‑daily (BID) doses were evaluated in the initial placebo‑controlled study in participants with moderate to severe AA [27], and 8 mg and 12 mg BID were also studied in subsequent placebo‑controlled pivotal trials in participants with severe AA [2,23,35]. The 4 mg BID dose was not advanced because a small phase 2 study found it ineffective and statistically indistinguishable from placebo over 24 weeks of therapy [27]. Using data from this very small study, the authors report that the 4 mg group “paradoxically had the highest percentage of patients achieving at least one adverse event (86.4%) while 8 mg recorded 81.6%, 12 mg BID 83.3%, and placebo 70.5%” [35]. In our view, there is nothing paradoxical about this observation - large placebo‑group adverse event rates and the random variation inherent to small samples commonly produce these patterns, which do not support causal inferences about dose‑related harm.

The report further claims that the “higher incidence” of adverse events in the 4 mg arm was “likely the reason this regimen (4 mg BID) was discontinued in later clinical studies” [35]. Neither pharmacologic principles nor the available evidence support this causal attribution. A more appropriate explanation for excluding 4 mg from later pivotal trials is its demonstrated inefficacy after 24 weeks of treatment [27]. Since this dose was neither advanced into pivotal studies nor approved, it is uninformative for clinicians selecting doses for severe AA.

The meta‑analysis also included deuruxolitinib 12 mg BID, a dose never approved and the subject of an FDA clinical hold on May 17, 2023, for “unfavorable benefit-risk” [2,36,37]. At the time of FDA approval of deuruxolitinib 8 mg, at least 14 nonfatal thrombotic events had been reported with the 12 mg dose, including deep vein thrombosis, bilateral pulmonary embolism, cerebral venous thrombosis, thrombophlebitis, and transient ischemic attack [36,37]. Therefore, approval was only sought for the 8 mg dose. These dose‑dependent events were not observed at the 8 mg approved dose. Inclusion of 12 mg data in a pooled analysis, particularly without explicit discussion of approval status, clinical hold, or differential safety risk, adds little value and may mislead dermatologists and other stakeholders. Finally, citing the comparable adverse event profiles of deuruxolitinib 4 mg and 8 mg in a small phase 2 study, along with the numerically higher headache rates at 12 mg relative to 8 mg in one of two pivotal studies, to suggest an “inverse dose-response relationship to certain adverse events, similar to the aforementioned point regarding the paradoxical effect of the lower dose,” is both inconsistent with the available evidence and clinically irrelevant. These doses are unlikely to be approved for the management of severe AA and offer no practical value to clinicians.

A recent network meta‑analysis by Gupta et al., submitted for publication in February 2025, compared placebo with 22 interventions, including oral JAK inhibitors, an injectable monoclonal antibody, and a phosphodiesterase‑4 (PDE4) inhibitor, administered by oral, injectable, and topical routes [38]. In some cases, the study pooled data from patients with moderate and severe AA, even though oral JAKI are approved only for severe disease (baseline SALT ≥50), and baseline SALT severity is known to affect the magnitude of treatment response.

Of the 22 active interventions included, only six correspond to approved drugs at approved doses [38]. The remainder reflect one or more of the following: (i) ineffective doses, (ii) unsafe doses, (iii) ineffective drugs, (iv) agents abandoned during clinical development, or (v) drugs discontinued for the AA indication, information that was publicly available well before publication. For example, the network meta-analysis reported 24-week SALT ≤20% responder rates for ritlecitinib at five dosing regimens as follows: 10 mg QD (1.69%), 30 mg QD (14.29%), 50 mg QD (23.39%), 200 mg QD for four weeks followed by 30 mg QD (22.31%), and 200 mg QD for four weeks followed by 50 mg QD (30.65%). However, ritlecitinib was approved 19 months earlier for severe AA only at the 50 mg dose. The remaining regimens of ritlecitinib, which lack global regulatory approval in any major market, are unlikely to inform clinical practice for the management of AA.

As in Kalantan et al.'s meta‑analysis, this network meta‑analysis includes deuruxolitinib 4 mg BID and 12 mg BID, neither of which is FDA‑approved (the 4 mg dose due to inefficacy; the 12 mg dose because of dose‑dependent nonfatal thrombotic events) [35-37]. Despite this, Gupta et al. reported that deuruxolitinib 12 mg BID for 24 weeks ranked as the most efficacious intervention for the primary endpoint (proportion achieving SALT ≤20) and the aspirational endpoint of SALT ≤10, an erroneous conclusion whose placement in the abstract risks outsized influence [38]. Inclusion of ineffective or unsafe regimens in a current network meta‑analysis undermines the clinical value of quantitative synthesis and risks producing misleading hierarchies for clinicians, guideline panels, and payors.

Finally, Qi and Li reported a systematic review and network meta-analysis evaluating the safety of oral JAKIs in the treatment of AA. Their analysis included both approved and unapproved drugs, as well as unapproved doses, ineffective doses, unsafe doses, and agents abandoned for the AA indication, many of which were publicly known to be nonviable well before publication. For example, the analysis incorporated baricitinib 1 mg QD and deuruxolitinib 4 mg BID (ineffective doses), deuruxolitinib 12 mg BID (an unsafe dose), and several drugs or regimens unapproved for AA as follows: deuruxolitinib 4 mg and 12 mg BID, tofacitinib, oral ruxolitinib, brepocitinib, and ritlecitinib 50 mg QD, preceded by four weeks at a loading dose of 200 mg QD [39].

In a recent study, deuruxolitinib at its only FDA‑approved 8 mg BID dose demonstrated robust efficacy in adults with severe AA [40], while sharing some of the same efficacy limitations of other JAKIs as follows: a SALT ≤20 response in roughly half of patients and safety concerns described for baricitinib and ritlecitinib [2,21,22,37]. Across analytic approaches, including Bayesian network meta‑analysis, multilevel network meta‑regression, and unanchored matching‑adjusted indirect comparisons, deuruxolitinib 8 mg BID consistently showed greater week‑24 efficacy for both SALT ≤10 and SALT ≤20 than baricitinib 2 mg and 4 mg QD and ritlecitinib 50 mg QD. Surface under the cumulative ranking (SUCRA) values corroborated these results as follows: deuruxolitinib 8 mg BID achieved the highest SUCRA for both endpoints, outperforming ritlecitinib and baricitinib (with baricitinib 4 mg showing intermediate efficacy). These findings support a provisional hierarchical preference favoring deuruxolitinib over baricitinib and ritlecitinib for appropriate patients with severe AA.

One potential limitation of deuruxolitinib is its BID dosing frequency, in contrast to baricitinib and ritlecitinib, which are administered QD, a convenience that can enhance adherence in chronic conditions [41-46]. Attempts to double the dose of deuruxolitinib to 16 mg and administer it QD yielded less favorable efficacy and safety outcomes, reinforcing the rationale for the approved 8 mg BID regimen [24]. Clinicians should weigh the convenience of QD dosing with baricitinib (2 mg and 4 mg) and ritlecitinib (50 mg) against the somewhat more favorable efficacy of deuruxolitinib 8 mg BID when discussing treatment options and adherence strategies with patients.

Regardless of the provisional efficacy hierarchy in favor of deuruxolitinib, interindividual differences in both efficacy and safety will undoubtedly necessitate an intraclass switch, that is, a change in treatment among the available oral JAKIs [26,31-33,47,48]. Additionally, when selecting an appropriate JAKI, clinicians must weigh not only therapeutic efficacy but also the safety profile and individual tolerability, pivotal determinants of long‑term treatment adherence and clinical success.

To improve transparency and usefulness, evidence syntheses should (i) prespecify the exclusion of doses never advanced into pivotal trials, or if such doses are included, analyze them in prespecified sensitivity or subgroup analyses; (ii) clearly report approval status and adverse regulatory actions (for example, clinical holds) alongside pooled safety estimates; (iii) avoid causal attributions from small, underpowered strata and instead present uncertainty with appropriate caveats; and (iv) conduct and report sensitivity analyses that remove unapproved or clinically held doses so clinicians, guideline panels, and payors can see how these choices affect both efficacy and safety estimates. In our view, implementing these practices would preserve the statistical advantages of meta‑analysis while protecting stakeholders from potentially misleading inferences drawn from small or unrepresentative dose groups.

Discussion

Some recent evidence syntheses in alopecia areata have pooled trials and dose groups that violate core meta‑analytic assumptions, reducing clinical utility. Major problems include inclusion of ineffective doses, unsafe doses, ineffective drugs, agents abandoned during clinical development, and drugs discontinued for the AA indication. In addition, omission of regulatory actions such as clinical holds further undermines the validity of pooled estimates. These practices have the potential to distort pooled efficacy and safety estimates, elevate unapproved regimens in ranking hierarchies, and invite inappropriate causal claims from small, underpowered strata. Small sample variability and large placebo‑group event rates commonly explain counterintuitive adverse event patterns without supporting dose-harm inferences. Pooling moderate and severe disease populations despite approvals for severe disease only further biases effect estimates. Collectively, these lapses risk producing misleading hierarchies that could misinform clinicians, guideline panels, and payors. These observations do not diminish the value of meta‑analytic methods when applied with appropriate safeguards, nor do they detract from the recent finding that deuruxolitinib 8 mg BID consistently outperforms baricitinib 2 mg and 4 mg QD and ritlecitinib 50 mg QD, supporting a provisional hierarchical preference for deuruxolitinib in appropriate patients with severe AA [40].

Conclusion

Network and conventional meta-analytic methods remain valuable for evaluating AA therapies, but must be applied with judicious study and dose selection. Implementing these safeguards will preserve the statistical advantages of meta-analysis while reducing the risk of distorted inferences from small or unrepresentative dose groups.

## References

[1] Hill AB (1937). Principles of Medical Statistics. https://www.jameslindlibrary.org/hill-ab-1937a/?utm.

[2] (2025). LEQSELVI (deuruxolitinib). https://www.accessdata.fda.gov/drugsatfda_docs/nda/2024/217900Orig1s000TOC.cfm.

[3] Zhou J, Liang L, Zhang H, Liu M, Zhu Z, Leng L, Li J (2025). Global burden of alopecia areata and associated diseases: a trend analysis from 1990 to 2021. J Cosmet Dermatol.

[4] Jeon JJ, Jung SW, Kim YH (2024). Global, regional and national epidemiology of alopecia areata: a systematic review and modelling study. Br J Dermatol.

[5] Hanson KA, Austin J, Clayton N (2025). Patient-reported psychosocial burdens and quality of life and work productivity impacts among patients with clinically distinct alopecia areata severity profiles. Adv Ther.

[6] Hanson KA, Vañó-Galván S, Messenger A (2025). Comparison of Dermatology Life Quality Index scores in adults and adolescents with alopecia areata. Dermatol Ther (Heidelb).

[7] Christou E, Lalagianni N, McSweeney SM (2025). Psychosocial burden and the impact of illness perceptions and stigma on quality of life, anxiety and depression in alopecia areata: results from the alopecia + me study. Br J Dermatol.

[8] Gregoire S, Biba U, Sanchez K, Mesinkovska NA, Waldman M, Anderson L, Mostaghimi A (2025). The financial and time burden of alopecia areata. J Dermatol.

[9] Abarca YA, Scott-Emuakpor R, Tirth J (2025). Alopecia areata: understanding the pathophysiology and advancements in treatment modalities. Cureus.

[10] Ramos PM, Anzai A, Duque-Estrada B (2025). II Consensus of the Brazilian Society of Dermatology for the treatment of alopecia areata. An Bras Dermatol.

[11] Mistry J, Sibbald C (2025). Clinical presentations and comorbidities of pediatric alopecia areata. Pediatr Dermatol.

[12] Kalil L, Welch D, Heath CR, Craiglow BG (2025). Systemic therapies for pediatric alopecia areata. Pediatr Dermatol.

[13] Harries MJ, Ascott A, Asfour L (2025). British Association of Dermatologists living guideline for managing people with alopecia areata 2024. Br J Dermatol.

[14] Rudnicka L, Arenbergerova M, Grimalt R (2024). European expert consensus statement on the systemic treatment of alopecia areata. J Eur Acad Dermatol Venereol.

[15] Seneschal J (2024). Alopecia areata: time for position statement to include new systemic therapeutic advances. J Eur Acad Dermatol Venereol.

[16] Meah N, Wall D, York K (2020). The Alopecia Areata Consensus of Experts (ACE) study: results of an international expert opinion on treatments for alopecia areata. J Am Acad Dermatol.

[17] Mateos-Haro M, Novoa-Candia M, Sánchez Vanegas G (2023). Treatments for alopecia areata: a network meta-analysis. Cochrane Database Syst Rev.

[18] King B, Guttman-Yassky E, Peeva E (2021). A phase 2a randomized, placebo-controlled study to evaluate the efficacy and safety of the oral Janus kinase inhibitors ritlecitinib and brepocitinib in alopecia areata: 24-week results. J Am Acad Dermatol.

[19] King B, Ohyama M, Kwon O (2022). Two phase 3 trials of baricitinib for alopecia areata. N Engl J Med.

[20] King B, Zhang X, Harcha WG (2023). Efficacy and safety of ritlecitinib in adults and adolescents with alopecia areata: a randomised, double-blind, multicentre, phase 2b-3 trial. Lancet.

[21] (2025). Olumiant (baricitinib) tablets, for oral use. https://www.accessdata.fda.gov/drugsatfda_docs/label/2022/207924s007lbl.pdf.

[22] (2025). Litfulo (ritlecitinib) capsules, for oral use. https://www.accessdata.fda.gov/drugsatfda_docs/label/2023/215830s000lbl.pdf.

[23] King B, Senna MM, Mesinkovska NA (2024). Efficacy and safety of deuruxolitinib, an oral selective Janus kinase inhibitor, in adults with alopecia areata: results from the phase 3 randomized, controlled trial (THRIVE-AA1). J Am Acad Dermatol.

[24] King B, Kempers S, Mesinkovska NA (2025). 62823 Optimization of deuruxolitinib dosing in adult patients with alopecia areata: results from a randomized, parallel-group, multicenter, phase 2 trial. J Am Acad Dermatol.

[25] Kwon O, Senna MM, Sinclair R (2023). Efficacy and safety of baricitinib in patients with severe alopecia areata over 52 weeks of continuous therapy in two phase III trials (BRAVE-AA1 and BRAVE-AA2). Am J Clin Dermatol.

[26] Kalil L, Craiglow BG, King B (2025). Successful treatment of alopecia areata with one JAK inhibitor after failure of other JAK inhibitors. JAMA Dermatol.

[27] King B, Mesinkovska N, Mirmirani P (2022). Phase 2 randomized, dose-ranging trial of CTP-543, a selective Janus kinase inhibitor, in moderate-to-severe alopecia areata. J Am Acad Dermatol.

[28] Piliang M, Soung J, King B (2025). Efficacy and safety of the oral Janus kinase 3/tyrosine kinase expressed in hepatocellular carcinoma family kinase inhibitor ritlecitinib over 24 months: integrated analysis of the ALLEGRO phase IIb/III and long-term phase III clinical studies in alopecia areata. Br J Dermatol.

[29] King B, Ko J, Kwon O (2024). Baricitinib withdrawal and retreatment in patients with severe alopecia areata: the BRAVE-AA1 randomized clinical trial. JAMA Dermatol.

[30] Peeva E, Guttman-Yassky E, Banerjee A (2022). Maintenance, withdrawal, and re-treatment with ritlecitinib and brepocitinib in patients with alopecia areata in a single-blind extension of a phase 2a randomized clinical trial. J Am Acad Dermatol.

[31] Yu M, Quan V, Li E (2025). Rational and efficacy of Janus kinase inhibitor switching for alopecia areata. J Am Acad Dermatol.

[32] Martin A, Sharma D, Mack S, Kelley K, Senna M (2025). Switching between Janus kinase inhibitors for treatment of alopecia areata. J Am Acad Dermatol.

[33] Peterson D, Powell M, King B (2021). Less is more? Failure of one JAK inhibitor does not predict failure of another one in a patient with alopecia areata. Dermatol Ther.

[34] Tsianakas A, Passeron T, Magnolo N (2025). Efficacy and safety of deuruxolitinib, an oral selective Janus kinase 1/2 inhibitor, in adults with alopecia areata: results from the THRIVE-AA2 phase 3, randomized, double-blind, controlled trial. J Am Acad Dermatol.

[35] Kalantan M, Bashrahil B, Aljuaid A, Bogari H, Samarkandy S, Jfri A (2025). Evaluation of the efficacy and treatment-emergent adverse events of deuruxolitinib for moderate to severe alopecia areata: a dose-ranging meta-analysis of 1,372 randomized patients. Front Med (Lausanne).

[36] Taylor NP (2025). Sun's concert hits bum note as FDA imposes partial hold 2 months after $576M buyout. https://tinyurl.com/6wvejuzb.

[37] (2025). Drug approval package: LEQSELVI, 8 mg tablets. https://www.accessdata.fda.gov/drugsatfda_docs/nda/2024/217900Orig1s000TOC.cfm.

[38] Gupta AK, Bamimore MA, Mirmirani P, Piguet V, Talukder M (2025). The relative efficacy and safety of monotherapies for alopecia areata: a network meta-analysis study. J Cosmet Dermatol.

[39] Qi Z, Li Y (2025). Safety of oral JAK inhibitors in treating alopecia areata: a systematic review and network meta-analysis. Front Pharmacol.

[40] Babul A, Mehta D, Soliman Y, Hussain M, Babul N (2025). Comparative efficacy of Janus kinase inhibitors indicated for severe alopecia areata: a Bayesian network meta-analysis and matching-adjusted indirect comparison. J Dermatol.

[41] Coleman CI, Limone B, Sobieraj DM, Lee S, Roberts MS, Kaur R, Alam T (2012). Dosing frequency and medication adherence in chronic disease. J Manag Care Pharm.

[42] Coleman CI, Roberts MS, Sobieraj DM, Lee S, Alam T, Kaur R (2012). Effect of dosing frequency on chronic cardiovascular disease medication adherence. Curr Med Res Opin.

[43] Kardas P (2005). The DIACOM study (effect of dosing frequency of oral antidiabetic agents on the compliance and biochemical control of type 2 diabetes). Diabetes Obes Metab.

[44] Laliberté F, Bookhart BK, Nelson WW, Lefebvre P, Schein JR, Rondeau-Leclaire J, Duh MS (2013). Impact of once-daily versus twice-daily dosing frequency on adherence to chronic medications among patients with venous thromboembolism. Patient.

[45] Lu X, Emond B, Morrison L (2023). Real-world comparison of first-line treatment adherence between single-agent ibrutinib and acalabrutinib in patients with chronic lymphocytic leukemia. Patient Prefer Adherence.

[46] Caldeira D, Vaz-Carneiro A, Costa J (2014). The impact of dosing frequency on medication adherence in chronic cardiovascular disease: systematic review and meta-analysis. Rev Port Cardiol.

[47] Huang J, Jian J, Li M (2026). Use of ritlecitinib in patients with alopecia areata refractory to tofacitinib or baricitinib: a single-center retrospective cohort study. J Am Acad Dermatol.

[48] Silva I, Khattri S (2025). Jak inhibitor intraclass switch in alopecia areata patients: a retrospective review of cases at an academic center. Arch Dermatol Res.

